# Recent acquisitions in the pathophysiology, diagnosis and treatment of disseminated intravascular coagulation

**DOI:** 10.1186/1477-9560-4-4

**Published:** 2006-02-21

**Authors:** Massimo Franchini, Giuseppe Lippi, Franco Manzato

**Affiliations:** 1 Servizio di Immunoematologia e Trasfusione – Centro Emofilia, Azienda Ospedaliera di Verona, Verona, Italy; 2 Istituto di Chimica e Microscopia Clinica, Dipartimento di Scienze Biomediche e Morfologiche, Università di Verona, Verona, italy; 3 Laboratorio di Analisi Chimico-Cliniche, Ospedale C. Poma, Mantova, Italy

## Abstract

Disseminated intravascular coagulation (DIC) is a disorder characterized by both acute generalized, widespread activation of coagulation, which results in thrombotic complications due to the intravascular formation of fibrin, and diffuse hemorrhages, due to the consumption of platelets and coagulation factors. Systemic activation of coagulation may occur in a variety of disorders, including sepsis, severe infections, malignancies, obstetric or vascular disorders, and severe toxic or immunological reactions.

In this review, we briefly report the present knowledge about the pathophysiology and diagnosis of DIC. Particular attention is also given to the current standard and experimental therapies of overt DIC.

## Background

Disseminated intravascular coagulation (DIC) is a disorder characterized by massive systemic intravascular activation of coagulation, leading to widespread deposition of fibrin in the circulation which can compromise the blood supply to various organs, thus contributing to multiple organ failure. At the same time, the consumption of platelets and coagulation proteins resulting from the ongoing coagulation may induce severe bleeding[1-6]. However, DIC is not a disease itself but is always secondary to an underlying disorder [7,8]. In fact, a variety of clinical conditions may cause systemic activation of coagulation. Table 1 lists the diseases most frequently associated with DIC. Bacterial infections, in particular septicemia, are the most common clinical conditions associated with DIC. There is no difference in the incidence of DIC in patients with Gram-negative or Gram-positive sepsis. Systemic infections by other micro-organisms, such as viruses and parasites, may also lead to DIC. The generalized activation of coagulation occurring in these cases is mediated by cell membrane components of micro-organisms (lipopolysaccharide or endotoxin) or bacterial exotoxins, such as staphylococcal α hemolysin, which cause a generalized inflammatory response through the activation of pro-inflammatory cytokines [9-13]. Severe trauma and burns are other conditions frequently associated with DIC [14,15]. Both solid and hematologic cancers may be associated with DIC, which can complicate up to 15 percent of cases of metastasized tumors or acute leukemia [16-18]. DIC is also a frequent complication (occurring in more than 50 percent of cases) of some obstetric conditions such as abruptio placentae and amniotic fluid embolism [19,20]. Finally, selected vascular disorders, such as giant hemangiomas and large aortic aneurysms, and severe toxic or immunological reactions (snake bites, drugs, hemolytic transfusion reactions and transplant rejection) can be associated with DIC [2,8].

**Table 1:** Clinical conditions associated with disseminated intravascular coagulation.

**Condition**	**Causes**
**Sepsis/severe infections**	Gram-positive bacteria, Gram-negative bacteria, spirochetes, rickettsiae, protozoa, fungi, viruses
**Trauma**	Polytrauma, neurotrauma, fat embolism, burns
**Malignancy**	Solid tumors, myeloproliferative/lymphoproliferative malignancies
**Obstetric complications**	Amniotic fluid embolism, abruptio placentae, placenta previa, retained dead fetus syndrome
**Vascular disorders**	Large vascular aneurysms, Kasabach-Merritt syndrome
**Organ destruction**	Severe pancreatitis, severe hepatic failure
**Toxic reactions**	Snake bites, recreational drugs
**Immunologic reactions**	Hemolytic transfusion reaction, transplant rejection

In the following sections we briefly discuss the current knowledge on the pathogenesis, diagnosis and treatment of DIC.

### Pathogenesis of DIC

Most of the recent advances in our understanding ofthe pathogenesis of DIC are derived from studies in animal models and humans with severe sepsis [6]. These studies have demonstrated that the systemic formation of fibrin observed in this setting is the result of the simultaneous coexistence of four different mechanisms: increased thrombin generation, a suppression of the physiologic anticoagulant pathways, impaired fibrinolysis and activation of the inflammatory pathway [1,8,21].

The systemic generation of thrombin has been shown to be mediated predominantlyby the extrinsic (factor VIIa) pathway. In fact, while the abrogation of the tissue factor/factor VIIa pathway resulted in complete inhibition of thrombin generation in experimental animal models of endotoxemia, the inhibition of the contact system did not prevent systemic activation of coagulation [22,23].

Impaired function of physiological anticoagulant pathways may amplify thrombin generation and contribute to fibrin formation [24]. Plasma levels of antithrombin are markedly reduced in septic patients as a result of a combination of increased consumption by the ongoing formation of thrombin, enzyme degradation by elastase released from activated neutrophils, impaired synthesis due to liver failure and vascular capillary leakage [6,7,25]. Likewise, there may be significant depression of the protein C system, caused by enhanced consumption, impaired liver synthesis, vascular leakage and a down-regulation of thrombomodulin expression on endothelial cells by pro-inflammatory cytokines, such as tumor necrosis factor (TNF)-α and interleukin(IL)-1β [26-28]. Moreover, the evidence that administration of recombinant tissue factor pathway inhibitor (TFPI) results in complete inhibition of endotoxin-induced thrombin generation suggests that tissue factor is involved in the pathogenesis of DIC [29,30]. Although no acquired or deficiency or functional defect of TFPI has been identified in patients with DIC, there is evidence that the inhibitor does not regulate tissue factor activity sufficiently in such patients[30].

As regards impaired fibrinolysis, experimental models of bacteremia and endotoxemia are characterized by rapidly increasing fibrinolytic activity, most probably due to the release of plasminogen activators from endothelial cells. However, this initialhyperfibrinolytic response is followed by an equally rapid suppression of fibrinolytic activity, due to the increase in plasma levels of plasminogen activator inhibitor type 1 (PAI-1) [31-33]. The importance of PAI-1 in the pathogenesis of DIC is further demonstrated by the fact that a functional mutation in the PAI-1 gene, the 4G/5G polymorphism, which causes increased plasma levels of PAI-1, was linked to a worse clinical outcome in patients with meningococcal septicemia [34].

Finally, another important mechanism in the pathogenesis of DIC is the parallel and concomitant activation of the inflammatory cascade mediated by activated coagulation proteins, which in turn can stimulate endothelial cells to synthesize pro-inflammatory cytokines. In fact, while cytokines and inflammatory mediators can induce coagulation, thrombin and other serine proteasesinteract with protease-activated receptors on cell surfaces to promote further activation and additional inflammation [35]. Furthermore, since activated protein C has an anti-inflammatory effect through its inhibition of endotoxin-induced production of TNF-α, IL-1β, IL-6 and IL-8 by cultured monocytes/macrophages, depression of the protein C system may result in a pro-inflammatory state [36].

Thus, inflammatory and coagulation pathways interact with each other in a vicious circle which amplifies the response further and leads to dysregulated activation of systemic coagulation [37]. Table 2  summarizes the most important mechanisms in the pathogenesis of DIC in sepsis.

**Table 2:** Pathogenesis of disseminated intravascular coagulation in sepsis.

**Mechanism**	**Pathophysiology**
**1) Increased thrombin generation**	Mediated predominantlyby tissue factor/factor VIIa pathway
**2) Impaired function of physiological anticoagulant pathway**	
a) Reduction of antithrombin levels	The result of a combination of increased consumption, enzyme degradation, impaired liver synthesis and vascular leakage
b) Depression of protein C system	The result of a combination of increased consumption, impaired liver synthesis, vascular leakage and down- regulation of thrombomodulin
c) Insufficienttissue factor pathway inhibitor (TFPI)	
**3) Impaired fibrinolysis**	Mediated by release of plasminogen activators from endothelial cells immediately followed by an increase in the plasma levels of plasminogen activator inhibitor type 1 (PAI-1)
**4) Activation of inflammatory pathway**	Mediated by activated coagulation proteins and by depression of the protein C system

However, there is evidence that various events, including the release of tissue material (fat, phospholipids, cellular enzymes) into the circulation, hemolysis and endothelial damage may promote the systemic activation of coagulation in severe trauma and burns through a mechanism similar to that observed in septic patients (i.e., systemic activation of cytokines)[14,15]. Nevertheless, there may also be specific variations in the pathogenesis if DIC due to different underlying disorders. For example, in some patients with cancer the initiation of coagulation activation is not only due to tissue factor expression on the surface of the malignant cells but in this case also involves a specific cancer procoagulant, a cysteine protease with factor X activating properties[38]. Patients with acute promyelocytic leukemia have a peculiar form of DIC characterized by a severe hyperfibrinolytic state associated with systemic activation of coagulation[17].

### Diagnosis of DIC

**Figure 1:**
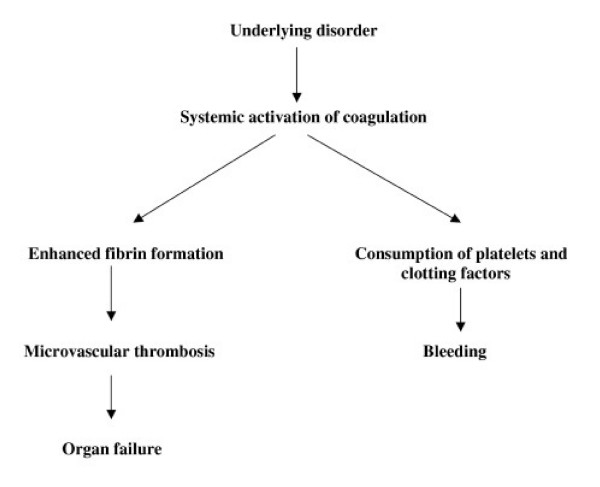
Clinical manifestations of coagulation abnormalities in disseminated intravascular coagulation.

**Figure 2:**
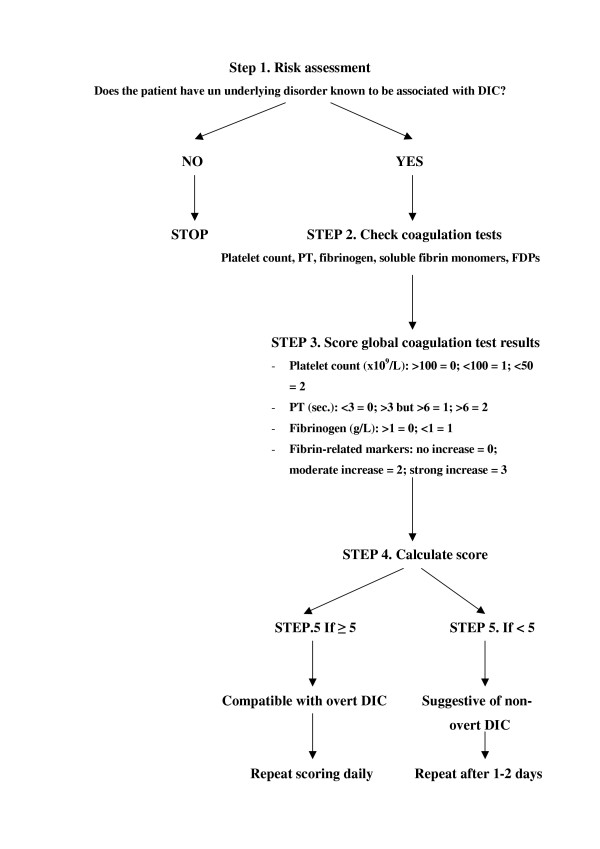
Five-step algorithm for the diagnosis of disseminated intravascular coagulation.

The most common clinical manifestations of DIC are bleeding, thrombosis or both, often resulting in dysfunction of one or more organs [39,40]. A schematic representation of the clinical manifestations of coagulation abnormalities in DIC is presented in Figure 1. Since no single laboratory test or set of tests is sensitive or specific enough to allow a definite diagnosis of DIC, in most cases the diagnosis is based on the combination of results of laboratory investigations in a patient with a clinical condition known to be associated with DIC [2,8]. The classical characteristic laboratory findings include prolonged clotting times (prothrombin time, activated partial thromboplastin time, thrombin time), increased levels of fibrin-related markers (fibrin degradation products [FDP], D-dimers), low platelet count and fibrinogen levels and low plasma levels of coagulation factors (such as factors V and VII) and coagulation inhibitors (such as antithrombin and protein C) [7,41]. However, the sensitivity of plasma fibrinogen levels for the diagnosis of DIC is low, since fibrinogen acts as an acute-phase reactant and its levels are often within the normal range for a long period of time. Thus, hypofibrinogenemia is frequently detected only in very severe cases of DIC [2,4]. On the other hand, FDP and D-dimer levels have a low specificity since many other conditions, such as trauma, recent surgery, inflammation or venous thromboembolism, are associated with elevated levels of these fibrin-related markers. Other, more specialized and useful tests, not available in all laboratories, include the measurement of soluble fibrin and assays of thrombin generation, such as those to detect prothrombin activation fragments F1+2 or thrombin-antithrombin complexes [42,43]. However, serial coagulation tests may bemore helpful than single laboratory results in establishing the diagnosis of DIC. A scoring system for the diagnosis of DIC, developed from a previously described set of diagnostic criteria [44], has been proposed by the Scientific Subcommittee on DIC of the International Society on Thrombosis and Haemostasis (ISTH) [45,46]. This system consists of a five-step diagnostic algorithm (see also Figure 2), in which a specific score, reflecting the severity of the abnormality found, is given to each of the following laboratory tests: platelet count (>100 × 10^9^/L = 0; <100 × 10^9^/L = 1, <50 × 10^9^/L = 2), elevated fibrin-related markers (e.g. soluble fibrin monomers/fibrin degradation products) (no increase = 0; moderate increase = 2; strong increase = 3), prolonged prothrombin time (< 3 sec. = 0; > 3 sec. but < 6 sec. = 1; > 6 sec. = 2), fibrinogen level (> 1 g/L = 0; < 1 g/L = 1). A total score of 5 or more is considered to be compatible with DIC. According to recent observations, the sensitivity and specificity of this scoring system are high (more than 90%) [46]. However, an essential condition for the use of this algorithm is the presence of an underlying disorder known to be associated with DIC [8]. Finally, a scoring system for diagnosing non-overt DIC has recently been proposed by the ISTH Scientific Subcommittee and validated by Toh and Downey who demonstrated its feasibility and prognostic relevance[47].

### Treatment of DIC

The heterogeneity of the underlying disorders and of the clinical presentations makes the therapeutic approach to DIC particularly difficult [2,48]. Thus, the management of DIC is based on the treatment of the underlying disease, supportive and replacement therapies and the control of coagulation mechanisms. The recent understanding of important pathogenetic mechanisms that may lead to DIC has resulted in novel preventive and therapeutic approaches to patients with DIC [49]. However, in spite of this progress, the therapeutic decisions are still controversial and should be individualized on the basis of the nature of DIC and the severity of the clinical symptoms [50-52]. The treatment for DIC include replacement therapy, anticoagulants, restoration of anticoagulant pathways and other agents [1,2,8]. Most of the clinical studies reported in the next paragraphs were conducted in patients with severe sepsis [53,54], a condition which usually leads to generalized activation of coagulation and thus represents an interesting model for the development of new treatment modalities. Table 3 summarizes the treatment modalities for DIC.

**Table 3:** Treatment modalities for disseminated intravascular coagulation.

**1) Replacement therapy**	- Fresh-frozen plasma
**2) Anticoagulants**	- Unfractionated and low-molecular-weight heparin
	- Danaparoid sodium
	- Recombinant hirudin
	- Recombinant tissue factor pathway inhibitor
	- Recombinant nematode anticoagulant protein c2
**3) Restoration of anticoagulant pathways**	- Antithrombin
	- Recombinant human activated protein C
**4) Other agents**	- Recombinant activated factor VII
	- Antifibrinolytic agents
	- Antiselectin antibodies
	- Recombinant interleukin-10
	- Monoclonal antibodies against TNF and CD14

#### a) Replacement therapy

The aim of replacement therapy in DIC is to replace the deficiency due to the consumptionof platelets, coagulation factors and inhibitors in order to prevent or arrest the hemorrhagic episodes [39]. Platelet concentrates and fresh frozen plasma (FFP) were, in the past, used very cautiously because of the fear that they might "feed the fire" and worsen thrombosis in patients with active DIC. However, this fear was not confirmed by clinical practice and nowadays replacement therapy is a mainstay of the treatment of patients with significant bleeding and coagulation parameters compatible with DIC. Transfusion of platelet concentrates at 1–2 U/10 Kg body weight should be considered when the platelet count is less than 20 × 10^9^/L or if there is major bleeding and the platelet count is less than 50 × 10^9^/L. When there is significant DIC-associated bleeding and fibrinogen levels are below 100 mg/dL, the use of FFP, at a dose of 15–20 mL/Kg, is justified. Alternatively, fibrinogen concentrates (total dose 2–3 g) or cryoprecipitates (1 U/10 Kg body weight) may be administered. However, FFP should be preferred to specific coagulation factor concentrates since the former contains all coagulation factors and inhibitors deficient during active DIC and lacks traces of activated coagulation factors, which may instead contaminate the concentrates and exacerbate the coagulation disorder.

#### b) Anticoagulants

The role of heparin in the treatment of DIC remains controversial [55-62]. In fact, although from a theoretical point of view interruption of the coagulation cascade should be of benefit in patients with active DIC [55], the clinical studies carried out so far have not been conclusive and indeed have often yielded contradictory results [56]. However, on the basis of the few data available in the literature, heparin treatment is probably useful in patients with acute DIC and predominant thromboembolism, such as those with purpura fulminans [2,56]. The use of heparin in chronic DIC is better established and it has been successfully employed in patients with chronic DIC associated with those diseases in which recurrent thrombosis predominates, such as solid tumors, hemangiomas, and dead fetus syndrome [61]. The role of heparin in the treatment of DIC associated with acute promyelocytic leukemia (APL) is another controversy, since some authors support its use whereas other studies failed to demonstrate its efficacy [57-59]. However, the use of heparin in this setting has declined in the last few years thanks to the introduction of all-trans retinoic acid therapy which has led to the reduction of APL-associated coagulopathy. Heparin is usually given at relatively low doses (5–10 U/Kg of body weight per hour) by continuous intravenous infusion and may be switched to subcutaneous injection for long-term outpatient therapy (i.e. for those patients with chronic DIC associated with solid tumors). Alternatively, low-molecular-weight heparin may be used, as supported by the positive results in both experimental and clinical DIC studies [60-63].

Experimental and clinical studies have also shown the potential role of danaparoid sodium, a low molecular weight heparinoid, in the treatment of DIC [64-66].

A newer anticoagulant agent with direct thombin inhibitory activity, recombinant hirudin (r-hirudin) [67,68], was recently shown to be effective in treating DIC in animal studies, although a study of the effect of this thrombin inhibitor in a sheep model of lethal endotoxemia showed no benefit [69]. Preliminary experimental human studies proved that this drug attenuated endotoxin-induced coagulation activation [70].

Since activation of the coagulation cascade during DIC occurs predominantlythrough the extrinsic pathway, theoretically the inhibition of tissue factor should block endotoxin-associated thrombin generation [71]. *In vivo *experiments in baboon models of lethal DIC showed that TFPI is a potent inhibitor of sepsis-related mortality [72]. De Jonge and colleagues [29] first demonstrated in humans that recombinant TFPI dose-dependently inhibits coagulation activation during endotoxemia. Recombinant TFPI was evaluated in a phase II randomized trial in patients with severe sepsis [73]. Although the study did not have the statistical power to demonstrate a survival benefit, it did show a trend toward a reduction in 28-day all-cause mortality together with an improvement in organ dysfunction in the group of patients treated with the recombinant TFPI. No evidence of a survival advantage was observed in patients with severe sepsis who received recombinant TFPI in a recent phase III large clinical trial[74].

Inhibition of the tissue factor/factor VIIa pathway is an another strategy that has been explored. Moons and colleagues demonstrated the efficacy of recombinant nematode anticoagulant protein c2 (NaPc2), a potent and specific inhibitor of the ternary complex between tissue factor/factor VIIa and factor Xa, in inhibiting coagulation activation in a primate model of sepsis [75]. Other authors have experimented with anti-tissue factor/factor VIIa antibodies in animal models with promising results [23].

#### c) Restoration of anticoagulant pathways

Since patients with active DIC have an acquired deficiency of coagulation inhibitors, restoration of the physiologic anticoagulation pathways seems to be an appropriate aim of the treatment of DIC [76]. Considering that antithrombin (AT) is the primary inhibitor of circulating thrombin, its use in DIC is certainly rational [77]. Recent studies in animals and humans with severe sepsis have demonstrated that antithrombin also has anti-inflammatory properties (reduction of C-reactive protein and IL-6 levels), which may further justify its utilization during DIC [78,79]. The administration of antithrombin concentrates infused at supraphysiologic concentrations was shown to reduce sepsis-related mortality in animal models [80]. Several small clinical trials have been conducted in humans, mostly in patients with sepsis-related DIC, and have shown beneficial effects in terms of improvement of coagulation parameters and organ function [81,82]. An Italian multicenter, randomized, double-blind study conducted in 1998 by Baudo et al. [83], evaluating the role of antithrombin in patients with sepsis or post-surgical complications, showed a net beneficial effect on survival in those patients receiving the concentrate. These findings were confirmed in 1999 by Levi et al. in their meta-analysis of all so far published human clinical trials of antithrombin treatment of DIC [3]. By contrast, a large randomized, controlled multicenter trial of supraphysiologic doses of AT concentrates conducted in 2144 patients with sepsis and DIC did not show a beneficial effect of antithrombin treatment [84]. However, a retrospective analysis of the same trial showed that the subgroup of patients who did not receive concomitant heparin had a potential benefit from antithrombin III in terms of mortality reduction[85].

Based on the fact that the protein C system is impaired during DIC some authors have investigated the therapeutic efficacy of exogenous administration of this protein in patients with DIC [86-98]. The infusion of activated protein C (APC) concentrates was shown to prevent DIC and mortality in an animal model of sepsis [99]. A study conducted in 1998 on patients with severe sepsis suggested a trend toward improved survival in the group treated with APC [88]. In a dose-ranging clinical trial, 131 patients with sepsis received recombinant human APC by continuous infusion at doses ranging from 12μg/Kg/hour to 30 μg/Kg/hour or placebo [89]. A 40 percent reduction in mortality was observed in those patients who received the higher doses of activated protein C. Similarly, another recent multicenter clinical trial [90] determined that treatment with recombinant human APC, given intravenously at a dose of 24 μg/Kg of body weight per hour, significantly reduced mortality in patients with severe sepsis, in spite of a higher rate of serious bleeding in the APC-treated group. A double-blind randomized trial compared the safety and efficacy of APC and unfractionated heparin in the treatment of DIC and concluded that the former improved DIC, and finally the survival, more efficiently than did heparin [91]. The recently published results of the trial conducted by the Human Recombinant Activated Protein C Worldwide Evaluation in Sepsis (PROWESS) Study Group showed a significant reduction in 28-day mortality and a quicker resolution of organ dysfunction in the group of patients with severe sepsis treated with APC [92,93]. These results were confirmed by the ENHANCE trial which also suggested that recombinant APC might be more effective is therapy is started earlier [94]. By contrast, a very recent trial on 2640 patients with severe sepsis and a low risk of death (defined by an Acute Physiology and Chronic Health Evaluation [APACHE II] score <25 or single organ failure) did not find a statistically significant difference in 28-day mortality rate between the placebo and APC-treated groups[95], suggesting that APC is of benefit only in patients at high risk of death from sepsis.Ongoing studies are focusing on the concomitant use of heparin in patients with DIC who receive activated protein C [8].

#### d) Other agents

Recombinant factor VII activated (rFVIIa) may be used in patients with severe bleeding who are not responsive to other treatment options. Bolus doses of 60–120 μg/Kg, possibly repeated after 2–6 hours, have been found to be effective in controlling refractory hemorrhagic episodes associated with DIC [100,101]. Antifibrinolytic agents, such as epsilon-aminocaproic acid or tranexamic acid, given intravenously at a dose of 10–15 mg/Kg/h, are occasionally used in patients resistant to replacement therapy who are bleeding profusely or in patients with disease states associated with intense fibrinolysis (prostate cancer, Kasabach-Merrit syndrome, acute promyelocytic leukemia) [102]. However, since these agents are very effective in blocking fibrinolysis, they should not be administered unless heparin has been previously infused in order to block the prothrombotic component of DIC. The use of these drugs in APL has declined in the last few years, given the efficacy of all-trans-retinoic-acid in preventing the majority of the hemorrhagic complications of this malignancy [8].

The advances in the understanding of the pathophysiology of DIC have resulted in novel preventive and therapeutic approaches to this disease. Based on the fact that tissue inflammation is a fundamental mechanism in DIC associated with sepsis or major trauma, some researchers have successfully employed the combined blockade of leukocyte/platelet adhesion and coagulation in a murine model by using antiselectin antibodies and heparin and have suggested the potential clinical use of such a strategy [103]. Based on the same rationale, other researchers have demonstrated that the administration of recombinant IL-10, an anti-inflammatory cytokine which may modulate the activation of coagulation, completely abrogated endotoxin-induced effects on coagulation in humans [104]. By contrast, the use of monoclonal antibodies against tumor necrosis factor has shown disappointing or at best modest results in septic patients[105-107]. More recently, Branger and colleagues[108]found that an inhibitor of p38 mitogen-activated protein kinase (MAPK), an important component of intracellular signaling cascades that mediate the inflammatory response to infectious and non-infectious stimuli, attenuated the activation of coagulation, fibrinolysis and endothelial cells during human endotoxemia. Finally, although studies using antibodies against the receptor for bacterial endotoxins (CD14) produced positive results[109], other studies using endotoxin antibodies failed to improve outcome[110,111]**.**

## Conclusion

Disseminated intravascular coagulation is a syndrome characterized by systemic intravascular activation of coagulation leading to bleeding (due to depletion of platelets and coagulation factors) and thrombosis(due to widespread deposition of fibrin in the circulation).

The diagnosis of DIC is usually made by a combination of routinely available laboratory tests, using a validated diagnostic algorithm.

In recent years, the mechanisms involved in pathological microvascular fibrin deposition in DIC have become progressively clear, resulting in novel preventive and therapeutic approaches to patients with DIC.
